# Biomechanical properties measured with dynamic Scheimpflug analyzer in central serous chorioretinopathy

**DOI:** 10.1007/s00417-024-06378-0

**Published:** 2024-01-29

**Authors:** Shuichiro Aoki, Ryo Asaoka, Keiko Azuma, Kohdai Kitamoto, Kohei Ueda, Tatsuya Inoue, Ryo Obata

**Affiliations:** 1 The University of Tokyo Hospital, 7-3-1 Hongo, Bunkyo-Ku, Tokyo, 113-8655 Japan; 2 Seirei Christopher University, Hamamatsu, Japan; 3 Yokohama City University, Yokohama, Japan

**Keywords:** Anterior segment optical coherent tomography, Central serous chorioretinopathy, Corneal biomechanical properties, Scleral thickness, Scleral stiffness

## Abstract

**Purpose:**

Recent evidence suggests that venous congestion at the vortex vein significantly contributes to the development of central serous chorioretinopathy (CSCR), and sclera is observed to be thicker in affected eyes. This study aims to investigate whether eyes with CSCR exhibit stiff corneas, measured using Corneal Visualization Scheimflug Technology (Corvis ST), which may serve as an indicator of scleral stiffness.

**Methods:**

This retrospective case–control study comprises 52 eyes from 33 patients diagnosed with CSCR and 52 eyes from 32 normal controls without CSCR. We compared biomechanical parameters measured with Corvis ST and anterior scleral thickness measured using anterior segment swept-source optical coherence tomography between the two groups.

**Results:**

Age, sex, axial length, intraocular pressure, and central corneal thickness showed no significant differences between the two groups (*p* > 0.05, linear mixed model). Three biomechanical parameters—peak distance, maximum deflection amplitude, and integrated inverse radius—indicated less deformability in CSCR eyes compared to control eyes. The stress–strain index (SSI), a measure of stiffness, and anterior scleral thickness (AST) at temporal and nasal points were significantly higher in the CSCR eyes. SSI and AST were not correlated, yet both were significantly and independently associated with CSCR in a multivariate logistic regression model.

**Conclusions:**

Eyes affected by CSCR have stiffer corneas, irrespective of thicker scleral thickness. This suggests that stiffer sclera may play a role in the pathogenesis of CSCR.
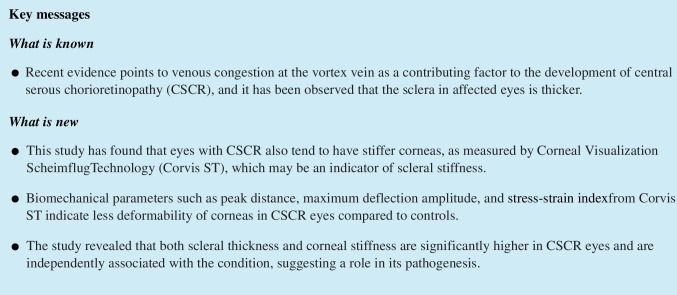

## Introduction

Central serous chorioretinopathy (CSCR) is a common retinal disorder characterized by serous retinal detachment in the macula, resulting in significant vision loss [1]. The pathophysiology is multifactorial, involving dysfunction of the choroid and retinal pigment epithelium, as well as systemic factors such as mental stress, inflammation, and hormonal imbalances. Notably, recent studies suggest that venous congestion at the vortex vein plays a vital role in the development of CSCR [2, 3]. This congestion induces changes in the choroid, such as venous dilation [4], intervortex venous anastomoses [5], and choroidal vascular hyperpermeability, which are similar to those observed in CSCR. Although the exact cause of this venous congestion in CSCR remains unclear, the association of a thickened sclera has been reported by several groups using anterior-segment optical coherence tomography (AS OCT) and ultrasound tomography [6, 7].

The sclera covers the majority of the ocular tunic, and its rigidity is associated with various factors such as axial length [8] and age [9]. We hypothesize that increased scleral stiffness, along with its thickness, increases the outflow resistance of the choroidal vein and contributes to the development of CSCR. Yet, direct measurement of scleral stiffness remains impractical in clinical settings.

In contrast, corneal stiffness can be directly measured using Corneal Visualization Scheimpflug Technology (Corvis ST, Oculus, Germany). This commercially available non-contact tonometer visualizes dynamic corneal responses to external pressure applied with an air pulse, captured by a high-speed Scheimpflug camera. Biomechanical parameters measured with Corvis ST have proven useful in managing various ocular diseases such as corneal ectasia [10] and glaucoma [11]. Given that the corneal and scleral stroma consist of similar extracellular matrix constituents, it is likely they possess common biomechanical properties [12, 13], suggesting that corneal stiffness could serve as a surrogate marker for scleral stiffness.

The purpose of this study was to compare the biomechanical properties measured with the Corvis ST between eyes with CSCR and controls. Additionally, we measured the anterior scleral thickness (AST) in these eyes using AS OCT and investigated its association with the stiffness-related parameter.

## Methods

In this case–control study, all of the eyes with CSCR and healthy eyes examined using the Corvis ST at the University of Tokyo Hospital during the period between June 2022 and March 2023 were retrospectively included in the current study. CSCR was diagnosed if serous retinal detachment or serous retinal pigment epithelium detachment in the macula was observed on optical coherence tomography, and at least one of the following two findings: leaks from retinal pigment epithelium on fluorescein angiography and choroidal vascular hyperpermeability on indocyanine green angiography. OCT angiography was also performed to rule out any potential neovascularization. Control eyes were those without any ocular pathology, including the pachychoroid spectrum disease or age-related macular degeneration, and they were matched for age, sex, axial length, and intraocular pressure (IOP) with the CSCR group. Eyes with the following features were excluded from both groups: patients with a history of steroid administration by any route; spherical equivalent refractive error less than − 6 diopter or more than 3 diopter; contact lens wearers; corneal diseases such as keratoconus and corneal ectasia; a history of any ocular surgery including corneal refractive surgery, cataract surgery, scleral buckling, and strabismus surgery; a history of panretinal photocoagulation or retinal degeneration; ocular hypertension of 25 mmHg or higher; glaucoma; and a history of any anti-glaucoma intervention including antiglaucoma medications, as antiglaucoma medications probably affect corneal biomechanics and scleral thickness [14, 15]. CSCR eyes with a history of photodynamic therapy were also excluded.

### Clinical data acquisition

Baseline demographic data such as age, sex, and axial length was collected from the medical charts. The spherical equivalence was calculated by adding half of the cylindrical power to the principal spherical power. Axial length was measured using the IOL Master ver. 5.02 (Carl Zeiss Meditec, CA, USA). Horizontal line scan images of macula were obtained with a spectral-domain OCT (Heidelberg Engineering GmbH, Dossenheim, Germany), and subfoveal choroidal thickness was measured manually using a built-in caliper. Choroidal thickness was measured as the perpendicular distance between the choroid-scleral junction and the retinal pigment epithelium–Bruch’s membrane complex. AST was measured using a swept source AS OCT (CASIA 2; TOMEY). AST and Corvis ST measurements were performed within 3 months interval in each eye.

### Anterior scleral thickness measurement using ASOCT

We owe much to the work of Imanaga et al. [7] for the method of AST measurement using AS OCT. We evaluated AST in 4 quadrants (superior, temporal, inferior, and nasal) with AS OCT. Scans with a diameter range of 12 mm, perpendicular to the limbus at 12, 3, 6, 9 o’clock positions, were obtained. For these measurements, participants were instructed to gaze in the direction opposite the quadrant being measured. The raster scan mode was used to obtain the desired B scan.

**Fig. 1:**
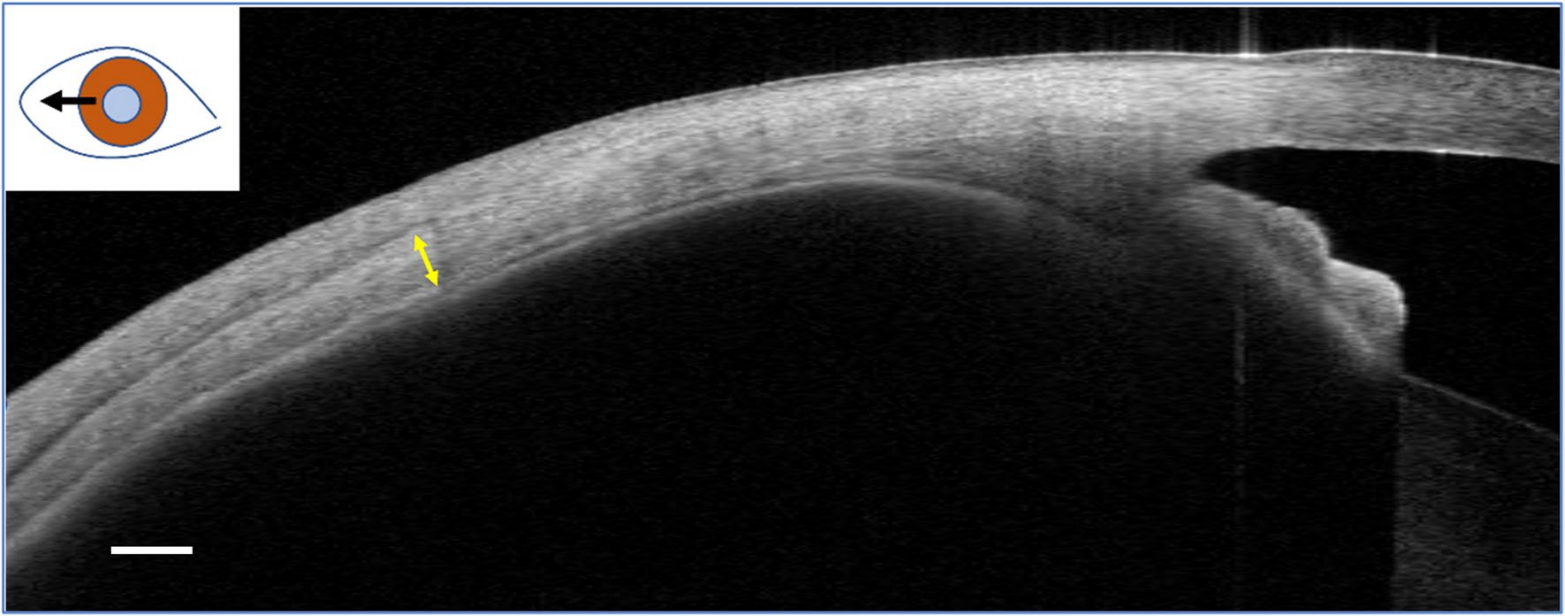
Cross sectional image of the temporal sclera obtained with anterior segment optical coherence tomography. The yellow line indicates the scleral thickness. The white bar at the bottom left represents 500 µm

On AS OCT scans, the sclera is visible as a hyperreflective band (Fig. 1). Anteriorly, the sclera was distinguished from the hyporeflective rectus muscles; posteriorly, the sclera was distinguished from the choroid appearing as a hyporeflective band. Previous studies [7, 16, 17] have used a constant distance from the scleral spur as the measurement point for AST. However, due to the variability in muscle attachment sites and the abrupt change in scleral thickness at these sites, we adjusted our measurement locations. For the nasal, temporal, and inferior quadrants, scleral thickness was measured at the anterior end of the distinct band corresponding to the rectus muscles. For the superior quadrant, scleral thickness was measured at a distance of 6 mm from the scleral spur, which included the contiguous tendon or scleral fibers continuous from the superior rectus muscle, since in many cases, the rectus muscle attachment was sufficiently posterior or not identifiable. Scleral thickness was measured perpendicular to the internal curvature of the sclera. Measurement was performed by a single, blinded examiner (S.A.). Quadrants for which AST could not be measured due to poor image quality were considered as missing data and excluded from the analysis.

### Corvis ST

**Fig. 2:**
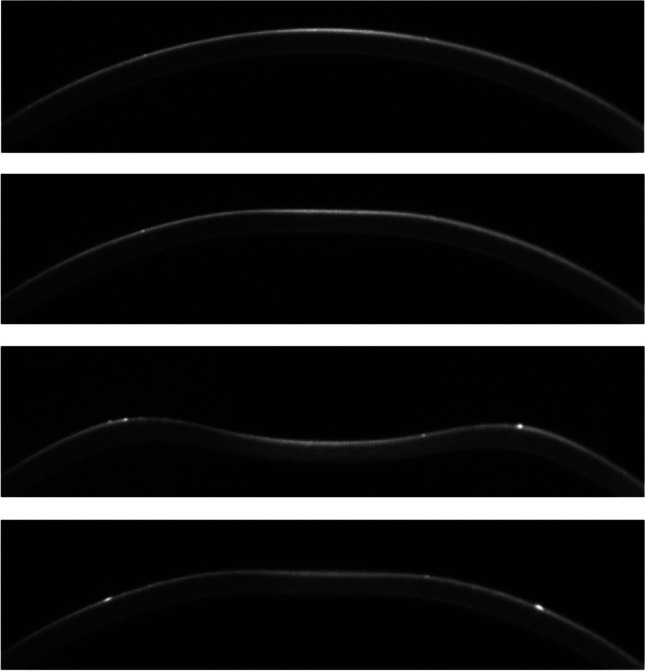
The different stages of corneal movement during Corvis ST measurement. The top image depicts the initial state before the application of an air puff, which is followed by the first applanation, highest concavity, and second applanation in sequential order from top to the bottom

Following the application of an air pulse onto the cornea, the Corvis ST device provides 140 images of the cornea in 30 ms using a high-speed Scheimpflug camera [18]. In response to the air puff, the cornea flattens (first applanation, A1), reaches its maximally concave shape (highest concavity, HC), and returns to the initial shape through flattening (second applanation, A2) (Fig. 2). The current Corvis ST software (version 1.6r2543) provides various morphological and mechanical parameters along with intraocular pressure (IOP) and central corneal thickness [19]. First/second applanation time (A1/A2 time) is the duration of the first and second applanations, while first/second applanation velocity (A1/A2 velocity) is the speed of the corneal apex at the first and second applanations. The “peak distance” is distance between the non-deformed peaks at HC, and the “deflection amplitude max” refers to the maximal displacement of the corneal apex from the baseline. The “integrated inverse radius” is the integrated curvature of the concaved cornea throughout the measurement, with higher values indicating less corneal deformation. The “pachyslope” represents the variation in the thickness of the cornea from the apex to the periphery [20]. Smaller pachyslope value indicates that the peripheral cornea is thin compared with the central part of the cornea. The “stiffness parameter A1” (SP-A1) is the ratio of the load on the cornea to its displacement at A1 [20]. Higher values of SP-A1 indicate stiffer corneas because more load is required to flatten the cornea. The “stress–strain index” (SSI) was developed to evaluate the material stiffness of the cornea [21]. In general, corneal stiffness largely depends on IOP and corneal geometry (ex. central corneal thickness) [19] due to the nonlinear relationship among IOP, corneal morphology, and the elastic modulus of the cornea [22]. SSI was developed based on a numerical model of physical behavior of the eye globe, whose material mechanical parameters were drawn from experiments using human cadaver eyes. Fitting data obtained with Corvis ST measurement to the model enables estimates of material stiffness of the cornea independent of IOP and corneal thickness. SSI represents the nonlinearity of the stress–strain relationship or material stiffness of the cornea [21]. SSI is normalized where the value of the average 50-year-old eye is 1. Higher SSI values indicate less corneal deformation and stiffer corneas.

Only reliable Corvis ST measurements, as indicated by the quality indicator “OK” displayed on the instrument monitor, were used for the present study.

### Statistical analysis

Mean values of clinical factors, Corvis ST, and AST were compared between CSCR and non-CSCR groups with the linear mixed models, whereby a patient was set as a random effect because one or two eyes of a patient were included. All values were expressed as mean ± standard deviation, and a *p* value of less than 0.05 was considered to indicate statistical significance. Correlation between SSI and AST averaged for four quadrants was assessed using the linear mixed model. Then, the optimal linear mixed model for the likelihood of CSCR was identified according to the second-order of the bias-corrected Akaike information criterion index (AICc) [23], from 2^2^ (= 4) patterns using SSI or average AST as covariates. A decrease in the AICc values suggests an improvement of the model. Likelihood ratio tests were performed to determine the significance of the covariate effects.

All data processing and analyses were performed using the statistical programming language, R (The R Foundation for Statistical Computing, Vienna, Austria).

## Results

Fifty-two eyes from 33 CSCR patients and 52 eyes from 32 non-CSCR patients were included in the study. Baseline characteristics of each group are shown in Table 1. Age, sex, axial length, IOP measured with Corvis ST, and central corneal thickness were similar among the groups (*p* > 0.05, linear mixed model). Subfoveal choroidal thickness was significantly thicker in the CSCR group than in the control group (*p* < 0.0001).

**Table 1:** Characteristics of CSCR and control groups. *p* value: linear mixed model

Parameter	CSCR (*n* = 52)	Controls (*n* = 52)	*p*
Age (years)	53.96 ± 8.23 (35.00 to 68.00)	55.06 ± 12.15 (29.00 to 80.00)	0.59
Male: female	33 (63%): 19 (37%)	31 (60%): 21 (40%)	0.84
Axial length (mm)	23.84 ± 0.90 (21.90 to 26.33)	24.10 ± 1.00 (21.99 to 26.19)	0.16
Intraocular pressure (mmHg)	15.51 ± 2.48 (9.50 to 22.00)	14.76 ± 2.55 (9.00 to 22.50)	0.25
Central corneal thickness (µm)	560.40 ± 42.24 (474.00 to 649.00)	556.23 ± 32.86 (466.00 to 632.00)	0.97
Subfoveal choroidal thickness (µm)	352.05 ± 92.32 (136 to 535)	241.96 ± 73.70 (76 to 408)	< 0.0001

Comparison of biomechanical parameters and AST is shown in Table 2. Among biomechanical parameters, CSCR group had a significantly lower magnitude of A2 velocity, peak distance, deflection amplitude max, and integrated inverse radius, and higher SSI (*p* = 0.0096, 0.042, 0.039, 0.045, and 0.0060, respectively, linear mixed model), the latter four of which indicate smaller deformation of the cornea in the CSCR group. Pachyslope was similar between the two groups (*p* = 0.86). CSCR group had a significantly higher AST at temporal and nasal quadrants (*p* = 0.032 and 0.019, respectively).

**Table 2:** Biomechanical parameters and AST in CSCR and control groups. *P* value: linear mixed model

Parameter	CSCR	Controls	*P*
A1 Time (ms)	7.50 ± 0.30 (6.81 to 8.32)	7.40 ± 0.31 (6.82 to 8.45)	0.23
A1 Velocity (ms)	0.15 ± 0.02 (0.11 to 0.18)	0.15 ± 0.02 (0.12 to 0.20)	0.23
A2 Time (ms)	21.82 ± 0.38 (20.66 to 22.62)	21.96 ± 0.45 (20.84 to 22.91)	0.13
A2 Velocity (ms)	-0.26 ± 0.03 (-0.34 to -0.19)	-0.28 ± 0.03 (-0.34 to -0.21)	0.0096
Peak distance (mm)	4.94 ± 0.26 (4.33 to 5.42)	5.10 ± 0.31 (4.30 to 5.68)	0.042
Deflection amplitude max (mm)	0.90 ± 0.09 (0.71 to 1.11)	0.96 ± 0.12 (0.75 to 1.21)	0.039
Integrated inverse radius	7.63 ± 1.06 (5.25 to 9.77)	8.19 ± 1.02 (6.09 to 10.19)	0.045
Pachyslope (µm)	44.76 ± 7.44 (27.92 to 63.67)	43.99 ± 7.58 (25.62 to 59.11)	0.86
SP-A1 (mmHg/µm)	109.81 ± 19.11 (61.15 to 149.82)	106.01 ± 18.27 (66.86 to 146.79)	0.46
SSI	1.23 ± 0.17 (0.90 to 1.53)	1.09 ± 0.23 (0.74 to 1.88)	0.0060
Superior AST (µm)	492.33 ± 53.86 (390.00 to 588.00)	485.02 ± 57.65 (330.00 to 593.00)	0.66
Temporal AST (µm)	403.14 ± 47.19 (292.00 to 530.00)	377.08 ± 45.86 (298.00 to 485.00)	0.020
Inferior AST (µm)	447.71 ± 63.20 (326.00 to 581.00)	427.49 ± 45.65 (337.00 to 519.00)	0.15
Nasal AST (µm)	435.23 ± 47.55 (323.00 to 519.00)	411.56 ± 42.18 (313.00 to 499.00)	0.032

*SP-A1* stiffness parameter applanation 1, *SSI* stress–strain index, *AST* anterior scleral thickness

**Fig. 3:**
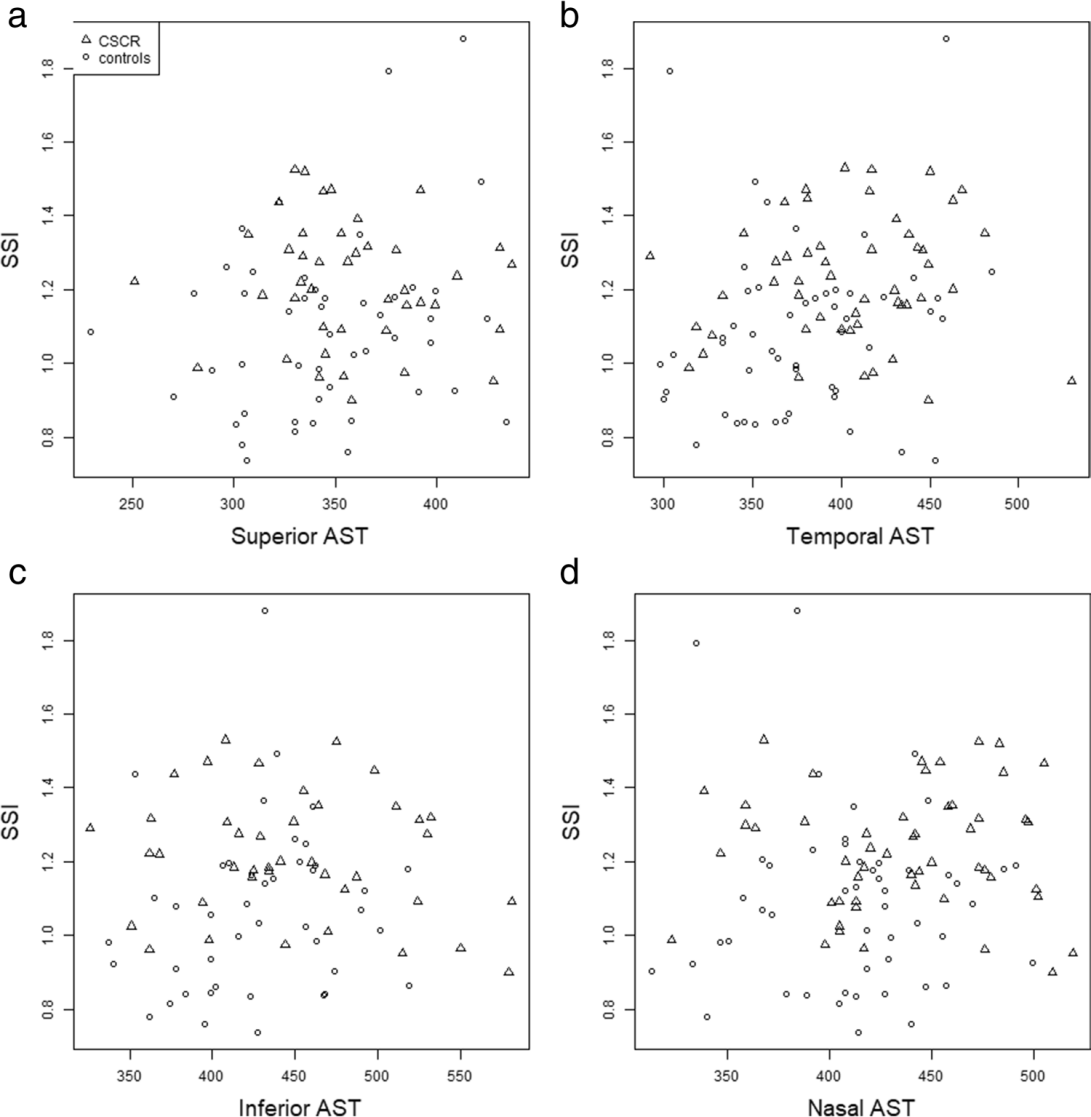
Scatter plots of SSI against AST at **a** superior, **b** temporal, **c** inferior, **d** nasal point. SSI, stress–strain index; AST, anterior scleral thickness

Scatter plots of SSI against AST are shown in Fig. 3. There was no significant association between SSI and AST in total, CSCR, or non-CSCR group (*p* = 0.21, 0.31, 0.12 for superior AST; *p* = 0.11, 0.69, 0.36 for temporal AST; *p* = 0.61, 0.37, 0.37 for inferior AST; *p* = 0.63, 0.87, 0.77 for nasal AST, respectively; linear mixed models).

An optimal logistic regression model to discriminate eyes with CSCR from control eyes was identified, in which covariates were selected from SSI and average AST. The equation for this model was as follows: (probability of CSCR) = 1/ [1 + exp( -52.60 + 52.35 × SSI + 5.64 x (average AST))]. AICc of this optimal model was 93.36, whereas that of logistic regression models with no covariates, only SSI, and only average AST, was 115.40, 97.29, and 105.12, respectively. Likelihood ratio test revealed both SSI and average AST significantly improved the model to discriminate the CSCR group from the control group (*p* = 0.00019 and 0.013, respectively).

## Discussion

In this study, we compared the biomechanical properties and anterior scleral thickness between CSCR and non-CSCR eyes. We found that CSCR eyes had significantly higher SSI values than non-CSCR eyes, suggesting stiffer cornea. Concordantly, other biomechanical parameters indicated that cornea was significantly less deformable in the CSCR group. In addition, temporal and nasal AST were thicker in CSCR eyes, although the stiffer cornea in these eyes was independent of this finding, and also the association between stiffer cornea and CSCR was independent from the thickness of AST.

Background factors, such as age, sex, axial length, intraocular pressure (IOP), and corneal morphology (represented by central corneal thickness and pachyslope), which could potentially affect the dynamic corneal response in Corvis ST measurements, were similar between the two groups. As such, their effects on the biomechanical parameters and corneal stiffness as a differential factor between the two groups were minimal. Nonetheless, significant differences were observed in peak distance, deflection amplitude max, and integrated inverse radius; corneal deformation was narrower and shallower in the CSCR group than in the non-CSCR group. This observation supports the hypothesis that CSCR eyes have a stiffer cornea. It also aligns with the finding that SSI was higher in the CSCR group than in the non-CSCR group. These results suggest that both the cornea and sclera are stiffer in CSCR eyes, given their shared biomechanical properties [12, 13]. The clinical implications of stiffer sclera in CSCR remain unclear. However, our hypothesis suggests a possible contribution to choroidal venous overload. A stiffer sclera may increase outflow resistance at the vortex veins, potentially playing a role in the development of CSCR [3]. Another possible consequence of a stiffer sclera could be its association with decreased permeability, thereby disturbing transscleral outflow. Increased resistance to transscleral outflow is a principal hypothesized mechanism in uveal effusion syndrome [24], which is characterized by ciliochoroidal effusion and serous retinal detachment. This syndrome, where a histologically altered sclera has been documented and the effectiveness of sclerotomy for particular subtypes is evident [25, 26], shares clinical characteristics with CSC and may have a common pathogenic mechanism involving scleral abnormalities [27, 28].

Previous studies have suggested a significant association between scleral stiffness and the extent of corneal deformation measured using Corvis ST. An experimental study using cadaver eyes demonstrated that the stiffening of the sclera with glutaraldehyde led to a less deformable cornea in Corvis ST measurements [29]. Another study found that a stiffer artificial ocular wall surrounding the cornea, compared with real sclera, resulted in a stiffer corneal response, implying that in vivo examinations may be influenced by scleral stiffness, in addition to corneal factors [30]. A different study showed that corneal deformation in response to external pressure (similar to Corvis ST measurements) decreased when the stiffness of the sclera was increased in finite-element models simulating the dynamics of the entire eyeball [31]. Also, in highly myopic eyes, which are characterized by less stiff sclera [8, 32], larger corneal depression [33], and lower SSI values [34] were observed than in emmetropic eyes. Thus, the smaller corneal deformation observed in the CSCR group in our study may suggest eyes with CSCR have stiffer sclera [21].

SP-A1 is another parameter of corneal stiffness, representing the secant elastic modulus of the cornea at the inward applanation of the cornea. In keratoconus, SP-A1 is significantly reduced compared with normal control eyes as early as in a subclinical stage without morphological change, indicating softer corneas [20, 35]. In the current study, there was no significant difference in SP-A1 between the two groups, in contrast to the significantly different SSI and biomechanical parameters related to maximal deformation between the two groups, and moreover, similar IOP levels in both groups. The reasons for these contradictory results are not fully understood, but these findings may suggest that SSI and SP-A1 represent different biomechanical properties of corneal stiffness. SSI is calculated based on values at the maximal deformation of the cornea, where the effect of the scleral stiffness is maximal [21], while SP-A1 is based on the corneal deflection amplitude at the inward applanation, which may reflect the stiffness of the cornea more directly [20]. Furthermore, SSI is more precisely corrected for IOP using whole globe numerical simulation than SP-A1, which means that SP-A1 may be more susceptible to IOP variance and would require a larger sample size to detect a difference. Further research is needed to shed light on these aspects.

We found significantly thicker temporal and nasal AST in the CSCR group than the control group, consistent with previous research reporting thicker sclera in CSCR [6, 7, 36]. However, there was no significant correlation between SSI and AST at any quadrant. Further, model selection revealed that both SSI and average AST were both significantly associated with CSCR. These findings suggest that corneal stiffness is independent from scleral thickness, and the associations of both variables with the development of CSCR are independent to each other.

Our study has limitations. First, it is a retrospective study and includes a small sample size. Future research should utilize larger sample sizes and prospective designs to further validate these findings. Second, the site of AST measurement relies on the patient’s muscle attachment site, which could lead to potential bias in measuring AST. This is because we found a large variation in rectus muscle attachment sites, with some anterior to 6 mm and others posterior to 6 mm. However, our method revealed that AST in CSCR eyes was higher than normal control eyes, replicating results of previous studies [7, 16, 17]. Third, AST has limited correlation with scleral thickness around the vortex vein, which is relevant in terms of the venous overload hypothesis. A histomorphometric study found that scleral thickness at the ora serrata had a significant correlation with that at the equator but not with the midpoint between the equator and the posterior pole [37]. Fourth, scleral stiffness around the vortex vein remains undetermined with the current methodology, a topic which is outside the scope of our study. Finally, additional investigation is also warranted to explore the correlation between biomechanical properties and other clinical aspects of CSCR, such as disease severity and the duration until resolution.

In conclusion, our study demonstrated that eyes with CSCR have a stiffer and less deformable cornea compared to control eyes, a characteristic that is independent of thicker sclera. This suggests that the stiffness of the ocular wall may play a role in the pathogenesis of CSCR.
